# Degradation and Bone-Contact Biocompatibility of Two Drillable Magnesium Phosphate Bone Cements in an In Vivo Rabbit Bone Defect Model

**DOI:** 10.3390/ma16134650

**Published:** 2023-06-28

**Authors:** Andrea Ewald, Andreas Fuchs, Lasse Boegelein, Jan-Peter Grunz, Karl Kneist, Uwe Gbureck, Stefanie Hoelscher-Doht

**Affiliations:** 1 Department for Functional Materials in Medicine and Dentistry, University Hospital of Wuerzburg, Pleicherwall 2, 97070 Wuerzburg, Germany; 2 Department of Oral and Maxillofacial Plastic Surgery, University Hospital of Wuerzburg, Pleicherwall 2, 97070 Wuerzburg, Germany; 3 Department of Trauma, Hand, Plastic and Reconstructive Surgery, University Hospital of Wuerzburg, Oberduerrbacher Street 6, 97080 Wuerzburg, Germany; 4 Department of Diagnostic and Interventional Radiology, University Hospital of Wuerzburg, Oberduerrbacher Street 6, 97080 Wuerzburg, Germany

**Keywords:** magnesium phosphate cement, phytic acid, drillability, bone replacement material

## Abstract

The use of bone-cement-enforced osteosynthesis is a growing topic in trauma surgery. In this context, drillability is a desirable feature for cements that can improve fracture stability, which most of the available cement systems lack. Therefore, in this study, we evaluated a resorbable and drillable magnesium-phosphate (MgP)-based cement paste considering degradation behavior and biocompatibility in vivo. Two different magnesium-phosphate-based cement (MPC) pastes with different amounts of phytic acid (IP 6) as setting retarder (MPC 22.5 and MPC 25) were implanted in an orthotopic defect model of the lateral femoral condyle of New Zealand white rabbits for 6 weeks. After explantation, their resorption behavior and material characteristics were evaluated by means of X-ray diffraction (XRD), porosimetry measurement, histological staining, peripheral quantitative computed tomography (pQCT), cone-beam computed tomography (CBCT) and biomechanical load-to-failure tests. Both cement pastes displayed comparable results in mechanical strength and resorption kinetics. Bone-contact biocompatibility was excellent without any signs of inflammation. Initial resorption and bone remodeling could be observed. MPC pastes with IP 6 as setting retardant have the potential to be a valuable alternative in distinct fracture patterns. Drillability, promising resorption potential and high mechanical strength confirm their suitability for use in clinical routine.

## 1. Introduction

Bony defects are a common and steadily increasing problem in virtually every surgical discipline that deals with the human skeleton. These defect situations can be caused either by iatrogenic interventions or in consequence of pathologies such as osteoporosis, trauma or primary intraosseous malignancies as well as bone metastases [1,2]. Treatment strategies vary according to the underlying pathology. Whereas smaller defect situations do not necessarily require a replacement of the deficient bone, larger defects which jeopardize the stability of the affected bone demand a proper reconstruction. In general, bone defects that cannot be stabilized by an internal implant like, for example, a plate osteosynthesis device or that cannot be healed by the body in the long term should be treated by filling the defect. The spectrum of bone reconstruction materials is vast and comprises autologous, allogenic, xenogeneic and synthetic bone replacement materials [3]. These groups can be subdivided further according to their origin and material properties and all of them have certain advantages and disadvantages. Available products include bone substitutes in the form of bone blocks or granules in various sizes. The gold standard of bone replacement is, despite many research efforts in this field, still autologous bone due to its unbeatable biocompatibility, resorbability, osteoconductivity, osteoinductivity, and its mechanical properties. It can be prepared in the same way as the autologous or synthetic bone substitutes, in the form of structural tricortical chips or as cancellous bone like the granules. However, its limited availability as well as the need for a second operation site to harvest the bone graft, typically from the iliac crest with the greatest gain in bone volume, are major drawbacks of autografts [4]. To avoid these drawbacks, synthetic bone replacement materials are being developed and refined. An almost unlimited disposability as well as their constant quality considering composition and structure are considered particularly beneficial in synthetic bone grafts. Application forms of synthetic bone replacement materials include, besides solid blocks or particulate granules, cementitious formulations [5]. The use of such bone cements is widespread in trauma and orthopedic surgery, and clinical applications can be found in the axial skeleton as well as in the extremities. Within the concept of augmented fixation, bone cements improve the structural strength and stability of the affected and mostly structurally compromised bone and therefore enhance the surrounding environment. In combination with standard fixation techniques, success rates, especially in the treatment of difficult fractures, can be improved [2]. In this context, drillability is a desirable feature for bone cements used in fracture treatment [6]. The decisive advantage of drillable cements is the complete filling of bony defects. In order to fulfill this requirement, bone cements need to withstand drilling and screw insertion without weakening the biomechanical stability of the bone substitute after an appropriate setting time [7]. Nevertheless, so far, there is no commercially available bone cement which meets the criterion of drillability for the purpose of augmented fixation. Among bone cements in general, calcium-phosphate (CaP)-based cements are considered to be very suitable as bone replacement materials due to their resemblance of the mineral phase of bone and their chemical and biological characteristics [8]. CaP bone cements show osteoconductivity, injectability, mouldability, self-setting capacity and great biocompatibility. On the other hand, there are limitations such as brittleness and poor mechanical strength, which impedes the use of CaP bone cements in load-bearing situations and makes them unsuitable for drilling applications [9]. Moreover, hydroxyapatite calcium phosphate cements, which constitute the current clinical standard, degrade and remodel to spongious bone only within years [10]. In the past years, magnesium-phosphate (MgP)-based bone cements have become a viable alternative in this field, where minerals such as struvite (MgNH_4_PO_4_·6H_2_O) or newberyite (MgHPO_4_·3H_2_O) are of special interest [11]. The suitability of MgP as an alternative to CaP-based bone cements derives mainly from its higher solubility and biocompatibility in vivo, which leads to increased resorption and new bone formation, which in turn is stimulated by the release of Mg ions [12,13]. MgP is mostly used in the form of cements. These have very good biomechanical properties, particularly with regard to their compressive and tensile strength, which in some cases exceed those of comparable CaP cements [14]. Due to their good mechanical properties, MgP cements (MPC) can be used in smaller non-load-bearing defects as well as in partially load-bearing defects [15,16]. Further advantages of these cements are their biocompatibility, degradability, good formability, and excellent adhesion in the graft bed [17]. Due to their alkaline pH value, MPCs are said to have antibacterial properties in some cases as part of their setting reaction [18]. A disadvantage of MPC also results from its setting, which in some cases is markedly exothermic and can lead to the release of potentially harmful ammonium ions. While the clinical handling of such cement pastes is relatively simple, the rapid setting time can limit their clinical usability [18]. Nevertheless, good strength values, an only moderate exothermic setting reaction and good and controllable absorbability speak in favor of the use of MgP bone cements in augmented fixation [7]. Notwithstanding the above, MPC formulations do not have a distinct drilling capability to meet the principle of augmented fixation [14]. In regard to this point, the addition of phytic acid (C_6_H_18_O_24_P_6_, inositol hexaphosphate, IP 6) can remedy the situation because it slows down the increase in viscosity within the cements, resulting in a more cohesive cement paste [19]. As previously demonstrated, the admixture of IP 6 to MPC pastes can lead to the production of a novel cement formulation of high mechanical strength and with drillable properties [7]. The addition of IP 6 to CaP cements improves their mechanical and flow properties, which is particularly relevant with regard to the use of such cements for minimally invasive applications or in a spatially restricted surgical site, as this allows cements to be introduced into bone defects even through narrow cannulas [20,21]. To evaluate the material properties of the IP 6-doped MPCs in vivo, two cement formulations with different IP 6 concentrations were implanted into an orthotopic defect model in the rabbit femur. Both cement compositions were chosen from a previous in vitro work where they demonstrated good clinical handling properties such as injectability, mechanical performance and pull-out strength of bone screws [7]. Although these results were obtained in a clinically relevant biomechanical test setup, they still can hardly mimic the complexity of a real bone defect situation. Cellular as well as physical accretion, degradation and remodeling processes during bone healing have a direct influence on the bone substitute materials used and their material properties. To quantify these influences, in this study, the IP6-doped MPCs tested in vitro were implanted in vivo for the first time ever in an orthotopic bone defect model in rabbits. This, in turn, is the basis for any further improvement of the IP6 MPCs used, adapted to the in vivo conditions. For material characterization, the cement pastes were first investigated with respect to their phase composition and porosity. The bone density and the density of the inserted bone substitutes were determined using peripheral quantitative computed tomography (pQCT), while the analysis of bone degradation and remodeling was performed using X-ray and cone beam computed tomography (CBCT). In addition, the biomechanical stability of both implanted bone substitutes and pure materials, in terms of the initial stiffness and compressive strength, was investigated. Consequently, the aim of this study was to evaluate two MPCs with different stoichiometries in terms of their bone-contact biocompatibility and degradability in vivo in order to obtain a valuable alternative to yet existing bone cements.

Our working hypothesis was that the 2 MgP cements used exhibited excellent bone-contact biocompatibility without inflammatory responses and almost completely remodeled into new bone within 6 weeks in vivo in a rabbit bone defect model.

## 2. Materials and Methods

### 2.1. Material Preparation and Characterization

Cement and adhesive compositions were prepared according to Table 1. Trimagnesium phosphate (Mg_3_(PO_4_)_2_) was prepared by sintering a mixture of 0.6 mol MgHPO_4_·3H_2_O and 0.3 mol Mg(OH)_2_ for 5 h at 1100 °C in a furnace (Oyten Thermotechnic, Oyten, Germany). The sintered cake was crushed, sieved to <355 µm and milled in a planetary ball mill (Retsch, Haan, Germany) with 500 mL jars and four 25 mm zirconia balls at 200 rpm for 1 h to a medium particles size d50 of 11.5 µm as measured with laser diffractometry (Microtrac, Haan Germany). Magnesium oxide was purchased from Magnesia GmbH (Magnesia GmbH, Lüneburg, Germany) and an IP 6 solution was obtained from Sigma Aldrich (Taufkirchen, Germany). Cement powders were sterilized by exposure to >25 kGy, while the IP 6 solutions were autoclaved at 121 °C for 20 min. All cement components (composition in Table 1) were mixed at a powder to liquid ratio (PLR) of 1.71 g/mL on a glass slab for 30 s. X-ray diffraction (XRD) patterns of the powder raw materials and the hardened cements were recorded in Bragg–Brentano geometry using a Bruker D8 Advance diffractometer with da Vinci design (Bruker, Karlsruhe, Germany) with Cu-Kα radiation (40 kV voltage, 40 mA current) in a 2-theta range from 20 to 40°. Qualitative evaluation of the diffraction pattern was performed using JCPDS references. Porosity measurements were undertaken by mercury intrusion porosimetry (Porosimeters Pascal 140 and 440, Thermo Fisher Scientific, Monza, Italy) by applying a gradual pressure ranging from 0.01 to 400 kPa. The compressive strength of the cements was evaluated by preparing cuboid samples of 12 × 6 × 6 mm in silicone rubber molds and hardening of the samples in PBS buffer for 24 h at 37 °C. Testing was performed on a universal mechanical testing machine (Zwick, Ulm, Germany) at a crosshead speed of 1 mm/min.

### 2.2. Animal Study Design and Surgery

To assess biodegradation and osseointegration of the MPCs, female New Zealand White Rabbits (Charles River, Sulzfeld, Germany) aged 105–111 days (2900–3300 g) were used as model organisms. The study was approved by the local authorities (permission filed by the Regierung von Unterfranken after passing the §15 commission as defined in the Animal Protection Act, file reference 55.2.2-2532-2-770) and was in compliance with international recommendations for care and use of laboratory animals (ARRIVE guidelines and EU Directive 2010/63/EU for animal experiments). For each cement variant, n = 6 was used. The number of animals needed was calculated with the help of G-power [22] using value 2 for Cohens d (means: difference between two independent means (two groups), analysis: a priori: compute required sample size).

On the day before surgery, the animals were weighed while awake and the weight-adapted preparation of the anesthetic drugs (Anesketin, Dechra, Aulendorf, Germany and Xylariem, Ecuphar GmbH, Greifswald, Germany) was carried out on the day of the operation. Pre-emptive analgesia (Metacam 0.35 mg/kg body weight, Boehringer Ingelheim, Germany, intramuscularly into the dorsal muscles) was performed before the surgical procedure. For surgery, the anesthetic was as well administered by intramuscular injection into the dorsal muscles, with onset of action in an average of 5–10 min. Gaseous anesthetics (Isoflurane, CP-Pharma, Burgdorf, Germany) were administered by mask to maintain anesthesia. Intraoperatively, the animals were monitored by a veterinarian. The implantation was carried out in a borehole defect in the femoral epiphysis to determine the degradation behavior and new bone formation in the volume.

After shaving the surgical area of the leg, it was washed with a disinfectant and covered with sterile drapes. Unilateral skin incision was performed in the area of the thigh (anterolateral approach) followed by a spreading preparation in the lateral intermuscular septum above the femoral condyle. The femur was then exposed over a length of approx. 2 cm and a drill hole (Ø = 5 mm) was made with a trephine drill, which reached into the medullary canal of the bone without drilling through the bone completely. Subsequently, the cements were inserted into this hole by using a spatula. The wound was then sutured in several layers (muscle, muscle fascia and skin) and spray dressing was applied. Postoperative analgesia was administered orally with Metacam (according to the veterinarian’s instructions).

For analyzing the outcome, animals were euthanized by a veterinarian using pentobarbital in overdose after anesthesia. Deep anesthesia was proved by the veterinarian. The femora with adherent tissue were placed into the mag stage of a Bruker Xtreme II imaging system (Bruker Corporation, Billerica, State, MA, USA) for taking X-ray images, followed by a high-resolution CBCT scan (Multitom RAX, Siemens Healthcare GmbH, Erlangen, Germany) and a measurement of the bone mineral density in a pQCT (Stratec XCT 2000, Stratec GmbH, Pforzheim, Germany).

### 2.3. Optical Assessment: Bone Mineral Density

**Figure 1:**
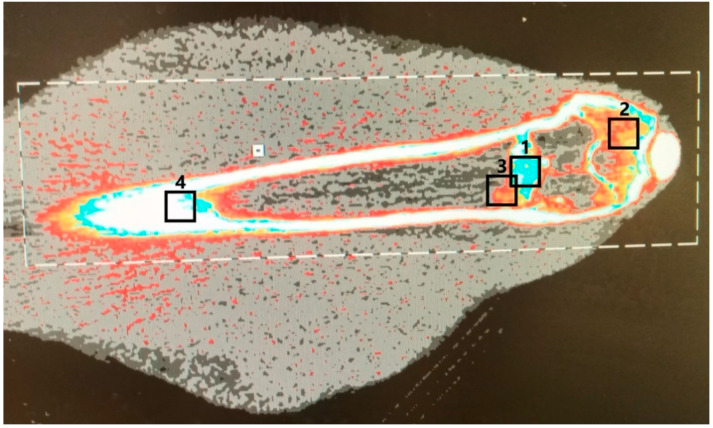
The 4 regions of interest were determined in a scout view and the bone mineral density (BMD) (mg/cm^3^) was measured for the bone substitute (1), the spongiosa (2), the transition areas between bone substitute and spongiosa (3) and for the corticalis of the diaphysis (4) in a pQCT, Stratec XCT 2000.

The bone mineral density in four different regions of the distal femoral bone was measured using a pQCT (Stratec XCT 2000, Stratec Medizintechnik GmbH, Pforzheim, Germany) in cooperation with the department of nuclear medicine. The measurements were performed directly after euthanization with the complete soft tissue around the femoral bone [23]. In a scout view of every bone, the four regions of interest (1: bone substitute, 2: spongiosa, 3: transition area between bone substitute and spongiosa, 4: corticalis of the diaphysis) were determined (Figure 1). These were defined by the senior Orthopedic surgeon (SHD). The bone substitute was located in the area of the highest density measurement on the distal femur (1), which was cylindrical in shape as seen intraoperatively during drilling. In the immediate area, the transition to the cancellous bone was defined as another region (3). On the distal femur, an area with visible cancellous structure was selected within the cortical bone (2). Finally, the cortical bone of the femoral diaphysis (white on the Figure 1), which is clearly visible in all planes, was used for comparison (4). The parameter of interest was the bone mineral density (BMD) of every region in mg/cm^3^.

### 2.4. X-ray and CBCT Imaging

**Figure 2:**
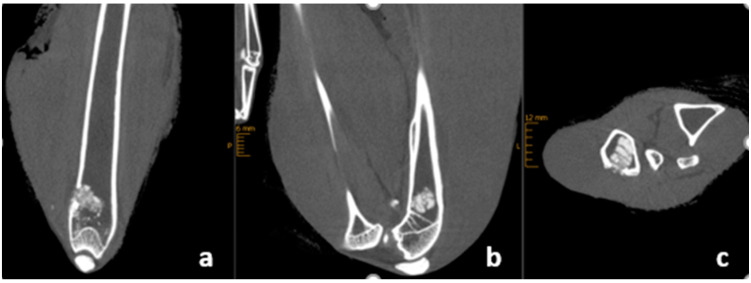
Radiological standard views reconstructed from a high-resolution cone-beam CT examination depict the implanted cements: coronal view (**a**), sagittal view (**b**), and in an axial view (**c**).

After euthanasia, the complete legs of the rabbits covered with the soft tissue underwent an imaging. Degradation and remodeling were evaluated analyzing the X-rays (Faxitron^TM^ Trident, Berlin, Germany) and high-resolution CBCT (Multitom RAX, Siemens Healthcare GmbH, Erlangen, Germany) (Figure 2) [24]. Qualitative evaluation was performed according to a score established by the CPC Registry multi-center study (CPC Registry: Observational Prospective Multi-center International Study on the Use of Injectable Calcium Phosphate Cements for the Treatment of Bone Defects in Adults. Graftys SA. ClinicalTrials.gov; Identifier: NCT02575352) (Table 2). Resorption was measured as a percentage in all three planes of the CT. Bone regeneration was assessed by looking for the presence of new bone formation at the edge of the bone cement, and structural reconstruction was assessed by looking for whether the bone cement had remodeled into a cancellous structure with formation similar to bone trabeculae. Radiographic unity of cement and femur means that the cement was remodeled in such a way that it could no longer be distinguished from the femur bone on conventional X-ray 6 weeks after implantation. Two clinically experienced observers evaluated the imaging and assigned the score points.

### 2.5. Biomechanical Evaluation and Histological Examination

#### 2.5.1. Sample Preparation

**Figure 3:**
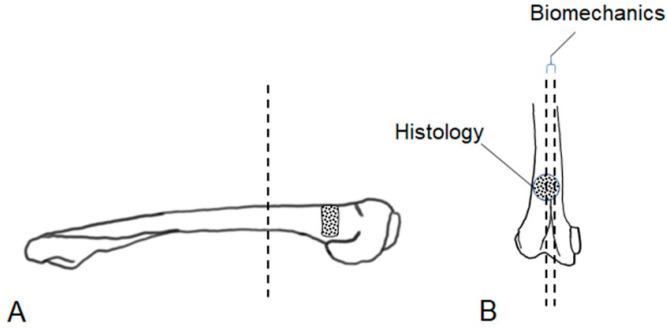
After dissection of the femoral bones, longitudinal slices were cut out for histological (**A**) and biomechanical (**B**) analysis by an Exakt band resaw.

After pQCT and imaging, the muscle tissue was removed, and the distal part of the femur (condyle plus implant area) was separated from the bone bearing the implant using an Exakt band resaw (Exakt Advanced Technologies GmbH, Norderstedt, Germany, Figure 3A). Then, this bone fragment was cut longitudinally with one half being used for histological analysis, while a central 3 mm thick slice (Figure 3B) was cut out from the other half for biomechanical evaluation [25].

#### 2.5.2. Biomechanical Evaluation

**Figure 4:**
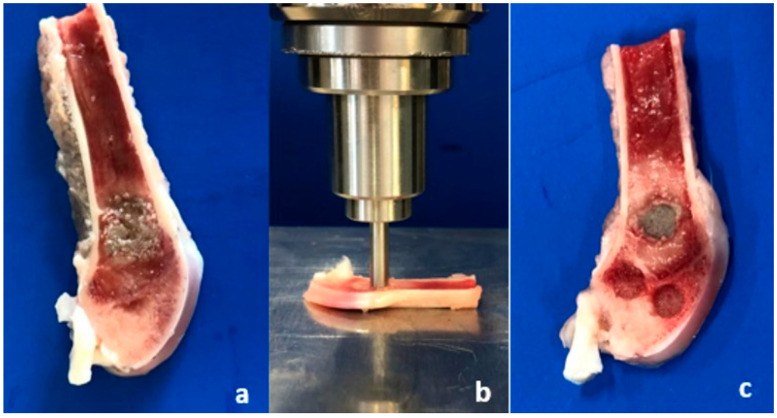
Directly after cutting a central 3 mm thick slice of the femoral bone (**a**; before testing) by a diamanted saw, the bones were biomechanically evaluated by an axial compression test (**b**) of 3 regions: the bone substitute and the ventral and dorsal spongiosa (**c**; impressions after testing).

Biomechanical tests were performed directly after cutting the femoral bones. In an axial loading test set-up (Figure 4b), an axial force with 1 mm/min was applied by an indentor (4 mm diameter) on the bone substitute and in 2 different regions of spongiosa (ventral/dorsal) (Figure 4c). Biomechanical evaluation was performed in a material testing machine Zwick Roell Z020 and the load-displacement curve was recorded by the testXpert^®^ II software (Zwick Roell, Ulm, Germany). The tests performed were load-to-failure tests (static tests). Endpoints of testing were defined as reaching a load of 400 N or a displacement of 1.5 mm. The initial stiffness was evaluated analyzing the slope of the elastic displacement in the load–displacement curve [25].

Besides the biomechanical tests of the femoral bone slices, static axial compression tests as pure material tests were performed. Therefore, cuboid samples of 12 × 6 × 6 mm were cured in a PBS bath (37°, 24 h) after 15 min of first setting. After 24 h of hardening, they were loaded in the material testing machine Zwick Roell Z020 with an axial load (1 mm/min) by an indentor. In order to analyze a possible reduction in biomechanical stability, a series with non-sterilized components and a second series of 12 samples with sterilized components were performed.

#### 2.5.3. Histological Examination

For histology, the specimens were fixed in a 10% formalin for 7 days and then transferred to Technovit 7200 (Heraeus Kulzer GmbH, Wehrheim, Germany) by dehydrating in an ascending series of Technovit 7100 followed by infiltration with Technovit 7200 in 5 steps, each step lasting 48 h. The samples were hardened in a mold by UV–light and then processed for cutting and grinding as described by Donath [26]. The slices were ground down to a thickness of about 20 µm (yield: three to four slices per sample). To analyze the implant’s tissue contact, the slices were stained using the Masson–Goldner–Trichrom staining procedure [27] and the contact area was determined using the software ImageJ (Rasband, W.S., ImageJ, U. S. National Institutes of Health, Bethesda, MA, USA).

### 2.6. Statistical Analysis

The statistical analysis of the experimental data obtained in this study was performed using IBM SPSS Statistics for Windows, version 28.0 (IBM Corp., Armonk, NY, USA) and in consultation with the Institute of Epidemiology and Biometry at Julius Maximilian University, Würzburg, Germany. Testing for normal distribution of the respective data collected in the course of the various measurements was performed using a Shapiro–Wilk test. As all data were normally distributed, analysis of a single outcome variable comparing several groups was performed using either a Student’s *t*-test or a one-way ANOVA followed by a Bonferroni post hoc test. To compare several measurements within a single group of material, either a paired-samples *t*-test or a repeated-measures ANOVA followed by a Bonferroni post hoc test was performed. Homogeneity of variance was tested accordingly using Levene’s test. The level of statistical significance was set at *p* < 0.05 for all comparisons. OriginPro software, version 2021b (OriginLab Corp., Northampton, MA, USA) was used for graphical presentation of the results.

## 3. Results

### 3.1. Material Characterization: Biomechanics and Porosimetry

**Figure 5:**
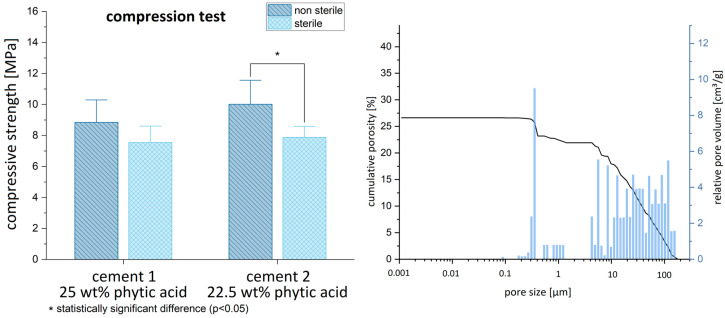
Compressive strength of non-sterile and γ-sterilized cements (**left**) and porosity characteristics of the cements in pure material tests (**right**) are shown (before implantation).

The MPCs displayed a compressive strength of approx. 9–10 MPa after their synthesis (cement 1: 8.84 ± 2.02; cement 2: 10.01 ± 2.17), which slightly decreased to 7.5 MPa following γ-sterilization with 25 Gy (cement 1 sterilized: 7.55 ± 1.47; cement 2 sterilized: 7.89 ± 0.99). No significant differences were found between the two IP 6 concentrations (non-sterilized: *p* = 0.23; sterilized: *p* = 0.56). The pore size distribution of both cements (Figure 5) showed the pores mostly in the range > 1 µm with a total porosity of approx. 27%. Only a few pores were found in the sub-micron region.

### 3.2. Phase Composition before and after Implantation

**Figure 6:**
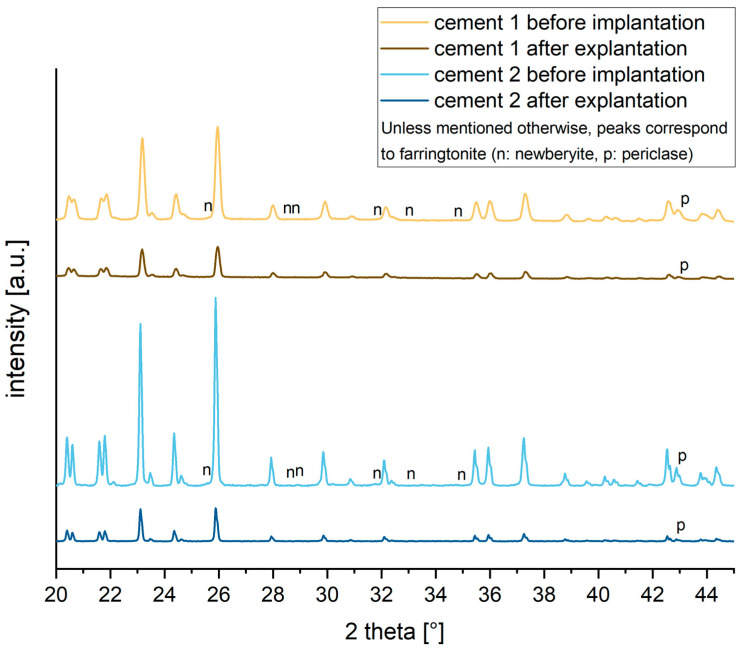
XRD pattern of drillable cements before and after implantation for up to 6 weeks.

Both MPCs before implantation consisted of a mixture of unreacted raw materials farringtonite and periclase with a minor fraction of newberyite (MgHPO_4_·3 H_2_O). The latter is formed as a by-product of the cement reaction by forming Mg^2+^–phytic acid complexes as previously shown by our group [19]. Following 6 weeks of orthotopic implantation, the overall intensity of the diffraction patterns decreased, while the phase composition was predominantly based on farringtonite with only very weak diffractions peaks of newberyite and periclase (Figure 6). The reduction in intensity is related to the small size of the explants such that there was not enough material to fill the whole sample holder.

### 3.3. Perioperative Animal Condition

At the day of surgery, the animals had a mean body weight of 3700 ± 240 g. After six weeks, their body weight increased substantially to 3989 ± 407 g (*p* = 0.04); no animal lost weight over the entire study period. All animals did behave well and showed no signs of suffering. Two animals had to be euthanized early due to complications: one animal developed a massive compartment syndrome and had to be euthanized at day 4 after surgery, and the other one showed a femoral fracture after 12 days.

### 3.4. Mineral Density

**Figure 7:**
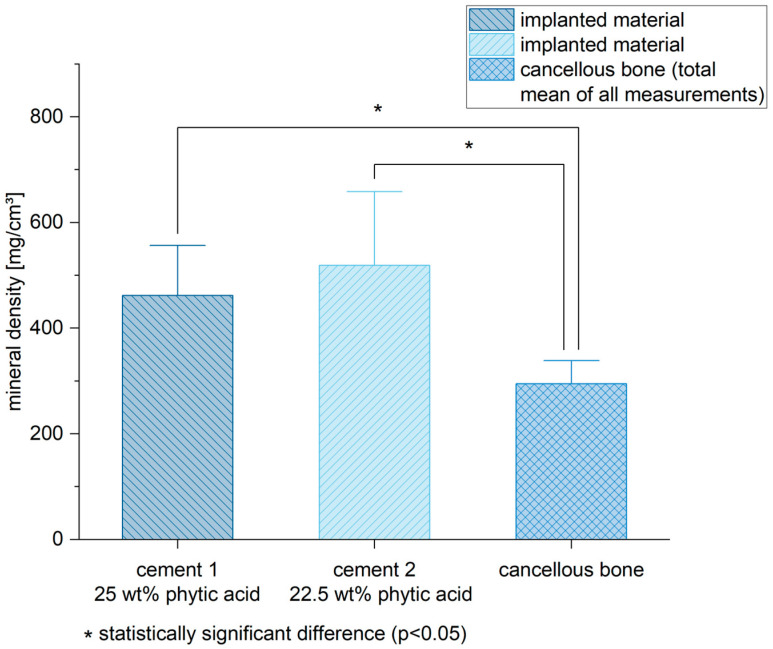
Both bone cements revealed a higher mineral density in the pQCT compared to the spongiosa. Significant differences are marked by *.

**Figure 8:**
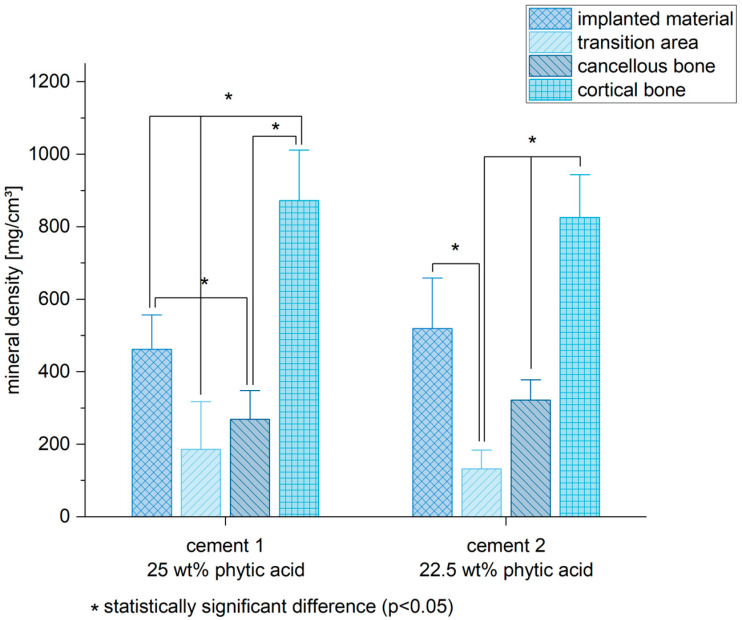
The cortical bone demonstrated the highest mineral density (MD) of all tested areas. Six weeks after the implantation, bone cements exhibited a higher MD compared to the cortical bone and the transition area between cement and spongiosa, in which the remodeling process of the cement is happening. Significant differences are marked by *.

Six weeks after the implantation, both bone cements still exhibited a higher mineral density in the pQCT compared to the spongiosa (mean of all measurements) (cement 1/spongiosa: *p* < 0.01; cement 2/spongiosa: *p* < 0.01) (Figure 7). Of all measured regions, the corticalis determined the highest mineral density compared to the other areas (Figure 8) (cement 1: *p* < 0.01 for all groups compared to corticalis except implanted material/corticalis: *p* = 0.02; cement 2: *p* < 0.01 for all groups compared to corticalis except implanted material/corticalis: *p* = 0.12).

### 3.5. Radiological Outcome

**Figure 9:**
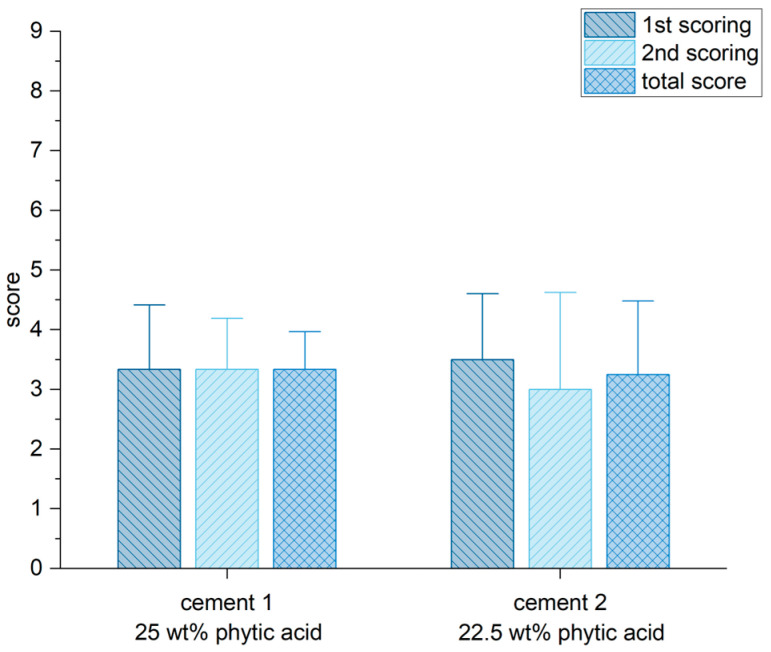
The highest score value for degradation, integration and remodeling was set by 9 points. Both cements used in this study reached around 3 points in the analysis of the imaging (CT-scans and X-rays). There was no difference in rating of both independent observers.

CBCT and X-ray scans showed a partial degradation and remodeling of the bone cements six weeks after implantation, but not a complete resorption and remodeling to spongiosa (highest score of 9 means a complete resorption and remodeling). No significant difference was ascertained between the independent ratings of both observers (Figure 9). Both cements individually reached 3.3 points from the possible maximum of 9 points.

### 3.6. Biomechanical Stability

**Figure 10:**
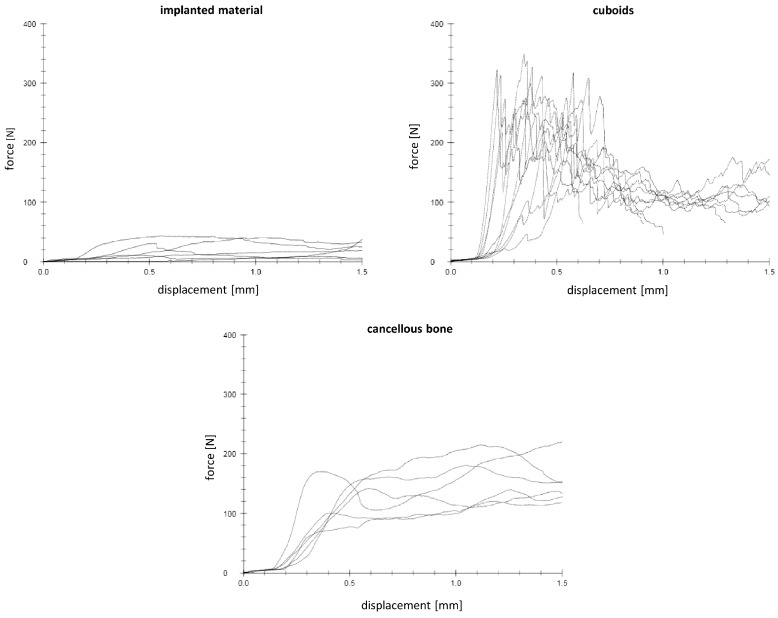
Force–displacement curves (cement 2) demonstrate the different behavior of the implanted material, cuboids and cancellous bone during the mechanical testing.

**Figure 11:**
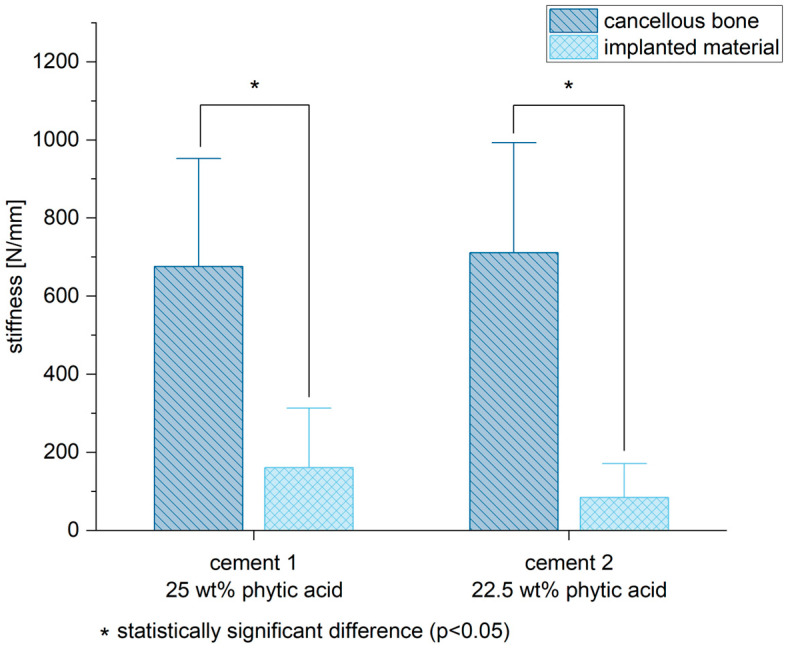
Both bone substitutes demonstrated biomechanically a lower stiffness compared to the cancellous bone. Significant differences are marked by *.

Both bone cements demonstrated a significant lower stiffness compared to the spongiosa (mean of the tests performed ventrally and dorsally of the cancellous bone) (cement 1/spongiosa: *p* = 0.01; cement 2/spongiosa: *p* < 0.01) (Figure 10 and Figure 11).

**Figure 12:**
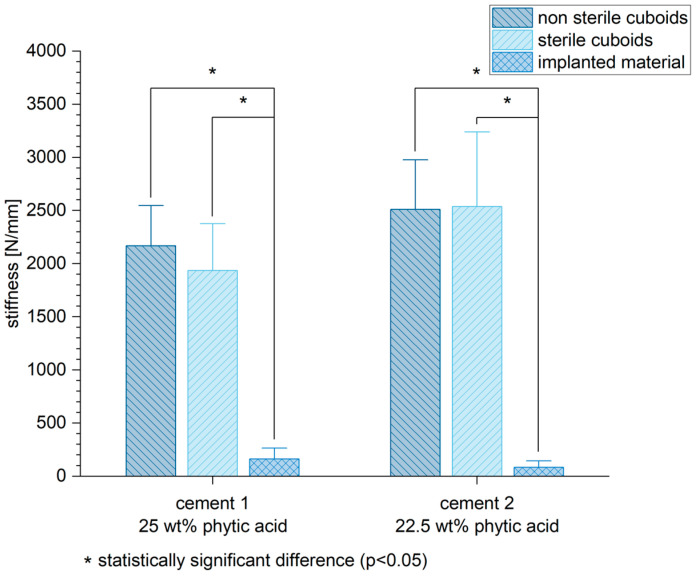
Six weeks after implantation, both bone cements revealed a lower stiffness in vivo compared to the pure material tests performed under either sterilized or non-sterilized circumstances. Significant differences are marked by *.

Besides the biomechanical tests performed on the bone slices, pure material axial compression tests of cuboid samples of the two different MPCs were performed to analyze a possible reduction in the biomechanical stability by the sterilization process of the components for intraoperative sterile use. The stiffness of the cuboid samples was either sterilized or non-sterilized significantly higher compared to the stiffness of the implanted cement after six weeks in vivo (Figure 12) (cement 1: non-sterile cuboids/implanted material *p* < 0.01, sterile cuboids/implanted material *p* < 0.01; cement 2: non-sterile cuboids/implanted material *p* < 0.01, sterile cuboids/implanted material *p* < 0.01).

### 3.7. Correlation of Stiffness and Mineral Density

**Figure 13:**
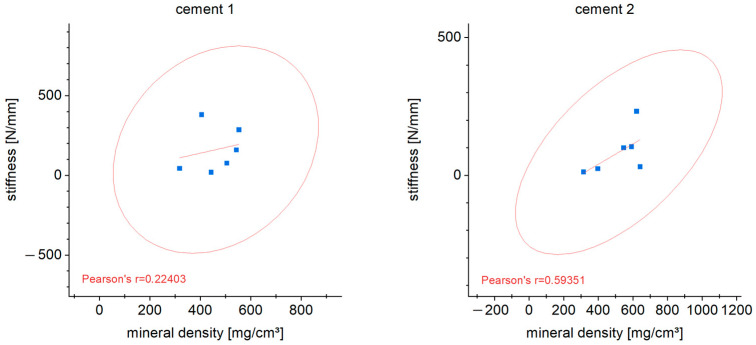
The correlation after Pearson between mineral density and stiffness of both bone cements and cancellous bone after 6 weeks in vivo is shown.

The correlation between stiffness and mineral density of the cements was shown in correlation analyses. A clear correlation (determined according to Pearson) was not found (cement 1: r = 0.22; cement 2: r = 0.59 (Figure 13).

### 3.8. Histological Examination

**Figure 14:**
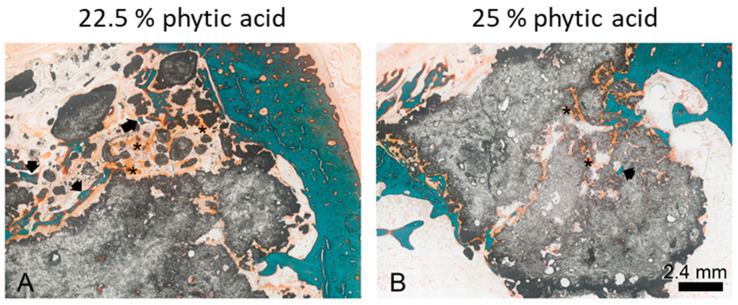
Masson’s Trichrome Stain of cut-grinded samples of bone cement implanted into rabbit femurs after six weeks. The cement was set with 22.5% phytic acid (**A**) and 25% phytic acid (**B**), respectively. Calcified bone is stained turquoise (arrows), osteoid appears orange (asterisks), the sample material turns out grey.

For differential staining of the cut grounded samples, the Masson–Goldner–Trichrom staining procedure [27] was used, which is ideal for staining connective tissue, muscle and bone. After this stain, different tissues are colored as follows: collagen—bright green, muscle—red, uncalcified bone (osteoid)—orange, calcified bone—turquoise. As shown in Figure 14 A and B, the bony structure of the corticalis is stained turquoise. Newly formed bone can be detected within cement particles as osteoid (asterisks) or already calcified (arrows). The bone implant contact was evaluated using the Image J software. Therefore, the length of the implant surface was measured in the images as a total (=100%), and then the contact area to calcified bone and osteoid, respectively, was determined. Figure 14 shows two representative slices of cements set with a 22.5% phytic acid (A) and a 25% phytic acid (B), respectively. In the figures, the grey sample material shows an irregular form with pores and some solitary particles. Due to the cutting plane, these may not be solitary in reality. These structures occur due to the application procedure of the pastes as there is no fixed abutment into which the pastes can be pressed. Only few direct contacts were detectable between bone tissue and the cements. In some areas, calcified bone was located amongst the material, but direct contact was mainly visible to the uncalcified osteoid. There were no signs of foreign body reaction nor inflammation.

**Figure 15:**
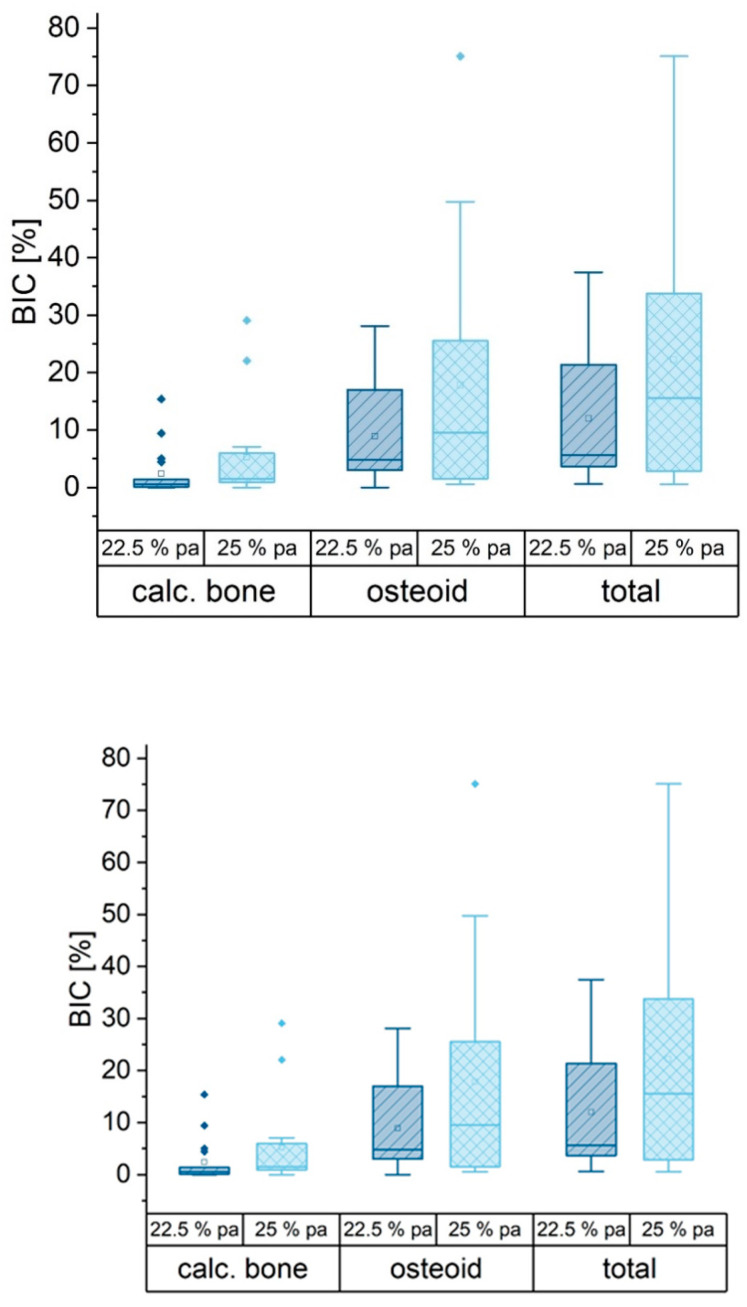
Box plot of bone implant contact given in % of the implant perimeter. More osteoid was formed around the implant compared to calcified bone (Light blue columns and diamonds: cement set with 25% phytic acid, dark blue columns and diamonds: cements set with 22.5% phytic acid).

For quantification of the bone implant contact (BIC), the contact areas were determined using the software Image J. The perimeter of the implanted material was measured as well as the part of calcified bone tissue and uncalcified osteoid. The part of osteoid in BIC was significantly higher than that of the calcified bone tissue. There was no significant dependence on the IP 6 concentration (Figure 15).

## 4. Discussion

The use of bone cements plays an increasingly important role for filling up bone defects instead of autologous bone transplantations, particularly in orthopedic surgery. Mineral cements are used alongside conventional osteosynthesis to stabilize selected fractures as part of the augmented fixation concept. The combination of stabilization technique and bone substitute is used frequently for fractures close to the joint or around tendon insertions on bones. In these areas, both high stability and good anchorage in the bone are required. Drillability as a property of the bone substitute is desirable here, as it allows complete filling of bony defects without weakening the stability of the bone filler [6]. MPCs represent a promising alternative to conventional hydroxyapatite cements on the market as bone substitutes [14,16,28]. In previous studies, the properties of MPCs with IP 6 as a liquid component were refined to such an extent that they excellently meet the necessary requirements of a bone substitute in terms of handling, processing time, viscosity, drillability, and biomechanical stability [7]. Ideally, in everyday clinical practice, the inserted bone cements are replaced over time by autogenous and functionally fully loadable bone in the course of fracture healing. Therefore, in the present study, two promising magnesium phosphate chemistry-based drillable bone cements were evaluated in a rabbit model with respect to their chemical, mechanical, and biological properties after implantation into the femoral bone defect. The results of the first analysis in vivo of these drillable MPCs are promising.

First of all, during reconvalescence, the animals did not show any signs of suffering indicating a proper healing of the defects. Also, after the discontinuation of analgesics, the animals were bright and curious and hopped around. Two animals had to be euthanized early. One showed a compartment syndrome which might be due to bleeding after the wound closure. The other one broke one leg by too proximal settlement of the bone defect with weakening of the femoral diaphysis, and also had to be euthanized early. This complication might be due to agility of the animal in combination with the not completely healed bone defect. The degradation and remodeling of the MPCs were evaluated based on the X-ray and CBCT examinations of the rabbit femora by the subjective assessment of two clinically experienced observers using a score with the criteria resorption of the bone substitutes, bone regeneration, radiological unity and structural reconstruction. When analyzing the present imaging, incipient degradation was evident for both MPC 22.5 and MPC 25, but without clearly discernible remodeling processes into cancellous bone. This may also be related to the resorption rate of MPCs, which may be faster than the formation of the newly formed bone. Within the scope of material properties of bone cements, resorption time plays an important role. Normally, the resorption of the bone graft should be in line with the rate of newly formed bone in order to ensure stability and volume of the bone. Therefore, the process of remodeling, not yet complete, to bone within six weeks after implantation is a satisfactory result for a clinical application. On the other hand, we suspected that remodeling to bone would be even more complete at 6 weeks, as formulated in our working hypothesis, than shown in our study. This imaging result was also reflected by histologic examinations.

Overall, the evaluation of the histopathological preparations of the MPCs showed an appealing picture with regard to their healing into the graft bed in vivo, especially for the formulations using a 25% phytic acid. Although there was a tendency for less surface contact between cement and calcified bone or osteoid compared to former studies [29], this can be partly attributed to the homogeneous application form of the cement pastes, which left relatively little space for tissue ingrowth in their central areas (Figure 14B). In the marginal areas, where presumably some degradation of the material had already taken place and the structure of the implants was accordingly more loosened, bony tissue was found adhering to the cements on several sites. One has also to consider that the particles in Figure 14A might appear due to fragmentation during the application process. A degradation cannot be proven by histology but rather assumed as it is not possible to obtain an image at t = 0 of the same specimen. In these samples, osteoid dominated over calcified bone, which was probably due to the time of explantation after six weeks. With regard to the bone implant contact, no difference was found in connection with the IP 6 concentration. There were no signs of inflammation or foreign body reaction in any of the specimens, again independent of IP 6 concentration. This result is very well in agreement with our hypothesis. Particularly in the marginal area of the MPC implants investigated here, a morphological picture similar to that of magnesium phosphate-based granulates and cement pastes without added IP 6 investigated in a previous study was observed [29]. Particularly with regard to the osteoid implant contact, the values were comparable, whereas the values in relation to the calcified bone implant contact were much lower, which is, once more, most likely to be due to the high density of the cement pastes inside the implant, but a delay effect of IP6 was not excluded. The formation of a fibrous capsule could not be observed.

In addition to histology and imaging, the MPCs displayed a lower biomechanical stability in terms of their initial stiffness compared to the cancellous bone (Figure 10). However, it is already known that the addition of IP 6 to calcium phosphate-based cements increases their mechanical performance [20]. The reduced mechanical stability of the MPCs could be, among other things, due to their partly very rapid resorption. Cements based on MgP chemistry showed clear signs of degradation in vivo already after six weeks of implantation, which could also affect their mechanical stability [29]. Although these cases involved cements with additional Ca admixture, it seems plausible that a higher Mg content, as in the present study, further increases the resorption potential of the bone cements. In order to evaluate the effects of the sterilization process on the cements, which is mandatory for implantation in vivo, and especially their biomechanical stability, pure material samples of MPC 25 and MPC 22.5 in sterilized and non-sterilized form were compared with the implanted samples after six weeks in vivo. Here, the pure material biomechanical compression tests, regardless of whether sterilized or non-sterilized samples were used, were clearly superior to the samples implanted in the animal model with regard to their initial stiffness. Although sterilization may have a weakening effect on the cements, stability was still high and did not differ significantly. Here, too, the degradation processes that took place in vivo could have been decisive for the reduced stability.

Considering the bone mineral density of the implanted MPCs, the pQCT measurements showed a relatively uniform picture for both stoichiometries (Figure 7 and Figure 8). No significant difference was observed between MPC 22.5 and MPC 25 after six weeks in vivo, whereas the bone mineral density of both cements was significantly higher compared to the local cancellous bone. This indicates a high structural stability of the cements centrally in the filling area and correlates with the histological and the biomechanical results. As expected, the local cortical bone showed the highest values, whereas the transition zone from bone cement to implant site showed the lowest values. This applied to MPC 22.5 as well as MPC 25 and can most likely be attributed to local bone remodeling induced in the transition zone between bone cement and the recipient site including osteoclast activation and therefore a reduction in local bone density.

Moreover, the pure material tests like the XRD analysis determined that the cements consisted mainly of farringtonite and periclase with a minor fraction of newberyite (MgHPO_4_·3H_2_O) (Figure 6). In a direct comparison of the two cement formulations, a greater amount of periclase was found in MPC 25. This can most likely be attributed to the higher concentration of IP 6, which provides more phosphate ions for the formation. With regard to porosity, both cements investigated showed similar values (Figure 5). However, MPC 22.5 showed a more homogeneous distribution of the relative pore volume and the pore sizes. Overall, pore diameters between 0.2 µm and 200 µm were found for both MPCs investigated. This corresponds approximately to the values described previously for other cements like struvite. Altogether, a lower concentration of IP 6 led to a lower porosity. In any case, the addition of IP 6 to cement pastes can significantly improve their injectability, leading to a low viscous cement that can be applied easily through a syringe without losing its cohesion [20]. This could be observed in our study, where cements were applied directly into the defect cavities without considerable resistance. Especially when regarding MPC formulations, the addition of IP 6 is known to lead to an increased stickiness which can further improve the mechanical properties of the bone cement amongst others in terms of their drillability [7,28]. Furthermore, it has been shown for IP 6-modified MPCs that the resistance of bone anchors implanted in bone to tensile loading can be increased by the addition of such IP 6 MPCs. The underlying mechanism was postulated to be the improved distribution of acting forces in the adjacent bone [30]. The drillability of cements can be a decisive advantage for bone cements in different fracture patterns. Unfortunately, the commercial cements that are available are not drillable without being destroyed and weakened by the drilling or screwing process. This is unfavorable because, for example, it has been shown that filling depression fractures of the tibial plateau with bone cements before conventional osteosynthesis results in higher postinterventional stability of the fracture at clinically relevant loads than filling after insertion of screws [6].

### Limitations

It should be considered that a small animal model such as the rabbit with a non-weight-bearing bone defect may not provide the same information content as tests on weight-bearing models in larger animals. One difference to the large animal model and therefore a different information benefit is the generally higher bone remodeling in rodents, which leads to a faster bone healing in these animals. Nevertheless, testing the newly developed materials in a small animal model first, it is well accepted for screening studies of new bone replacement materials that the rabbit model, as we used it in this study, is a valuable tool to initially evaluate parameters like bone regeneration capacity and material behavior in vivo [31,32]. However, the small sample size in experimental animal studies, in this present project with six samples per group, is always to be seen as a limitation, because the groups have to be kept as small as possible due to ethical framework conditions and limitations in animal husbandry. Therefore, this study should rather be seen as a pilot study for the first classification of the presented experimental drillable MgP cements. Due to the limited sample size, another limitation of the study design is that a small central slice of the femur bone used for the biomechanical analyses was not available for histological evaluation.

The cements tested in this study are characterized, among other things, by their drillability and thus have a unique feature in contrast to other cements. A limitation of the animal study is that this particular property was not tested. However, the good bone-contact biocompatibility and not the primary mechanical properties of the cements has been the focus of the cements presented here. Further investigations are necessary to characterize the MgP cements in greater detail, for example, under load-bearing conditions in a sheep model. Likewise, the aspect of drillability should be considered in further animal testing.

## 5. Conclusions

The present study has shown that magnesium phosphate cements modified with phytic acid can be successfully implanted in animal models without major loss of their material properties. Despite a reduced stiffness compared to native bone tissue, their drillability makes the two cement formulations investigated here an interesting alternative in selected orthopedic fracture patterns, for example, in the context of augmented fixation. Their previously proven drillability can contribute to increasing the primary stability of the fracture immediately after fracture treatment, while their resorbability means that they can be effectively replaced by the body’s own bone during the healing process.

## Figures and Tables

**Table 1:** Composition of bone cements for the current study.

	Cement 1	Cement 2
Powder	7.929 g Mg_3_(PO_4_)_2_ 0.643 g MgO	7.989 g Mg_3_(PO_4_)_2_ 0.583 g MgO
Liquid	25% IP 6	22.5% IP 6

**Table 2:** Evaluation score of the imaging (X-rays and cone-beam RAX slices) according to the clinical multi-center study following up the resorption of calcium phosphate bone substitute (CPC Registry study Graftys SA. ClinicalTrials.gov; Identifier: NCT02575352, accessed on 15 July 2015).

**Resorption of the Bone Substitute**	
None	0
Resorption of 0–25% of the material	1
Resorption of 25–50% of the material	2
Resorption of 50–75% of the material	3
Resorption of 75–100% of the material	4
**Bone regeneration**	
None	0
Evidence of bone regeneration	1
**Radiological unity**	
None	0
Possible unity	1
Radiological unity	2
**Structural reconstruction**	
None	0
Reconstruction in progress	1
Completed reconstruction	2
**Maximum points**	**9**

## Data Availability

The data presented in this study are available on request from the corresponding author.
